# Supplementary material for: Complexome profiling of the Chlamydomonas psb28 mutant reveals TEF5 as an early PSII assembly factor
Source: Plant Cell. 2025 Mar 17;37(6):koaf055. doi: 10.1093/plcell/koaf055 (PMC12164590; doi:10.1093/plcell/koaf055)
Supplement: koaf055_Supplementary_Data [file koaf055_supplementary_data.zip › psb28_interactive.zip!/plots/intensity/qualityPlots/peptides/_Cre07.g349200.t1.2.html]

### Cre07.g349200.t1.2

Trivial name:   
SeqCoverage [median]: 15.10  
Mass: 16.32 kDa  
  
 

| Sequence | MissedCleavage | Mass [Da] | wtIntensity | muIntensity | Score |
| --- | --- | --- | --- | --- | --- |
| ATTTTTTTTATTK | 0 | 1298.66 | 8451.46 | 3833.79 | 86.34 |
| ATTTTTTTTATTKK | 1 | 1426.75 | 50060.40 | 35869.12 | 113.76 |
| ENNPEMSR | 0 | 975.41 | 8390.63 | 10963.30 | 138.83 |
| KATTTTTTTTATTKK | 2 | 1554.85 | 627.35 | 1578.11 | 118.08 |
|  |  |  | 67529.8 | 52244.3 |  |
